# A Novel Yeast Surface Display Method for Large-Scale Screen Inhibitors of Sortase A

**DOI:** 10.3390/bioengineering4010006

**Published:** 2017-01-24

**Authors:** Lin Wu, Huijun Li, Tianle Tang

**Affiliations:** School of Tropical and Laboratory Medicine, Hainan Medical University, Haikou 571101, China; woshiwulin728@163.com (L.W.); lhj29@163.com (H.L.)

**Keywords:** sortase A, yeast surface display, LPETG motif, berberine chloride

## Abstract

Fluorescence resonance energy transfer substrates of sortase A are too expensive to be used to roughly screen high-throughput sortase A inhibitors. This makes therapeutic strategies difficult to realize in a clinical therapeutic use. Instead, we design here an LPETG-EGFP (leucine, proline, glutamic, threonine and glycine-enhanced green fluorescence) protein displayed on a yeast surface as a substrate by adaptively reducing the cost. We do this by optimizing the induction conditions of sortase A expression in *Escherichia coli* DE3(BL21) and catalyzing LPETG proteins, which are displayed on surface of *Pichia pastoris*. Different expression conditions of sortase A include: induction temperature (22 °C, 28 °C, 37 °C and 40 °C), induction time (4 h, 5 h, 6 h and 7 h) and induction concentration of isopropyl β-d-thiogalactoside IPTG (0.25 mmol/L, 0.5 mmol/L, 1 mmol/L, and 2 mmol/L). The fluorescence change of the LPETG-EGFP protein on the surface of *P. pastoris* over time was detected by flow cytometry and fluorescence spectrophotometry, and then the sensitivities of the two methods were compared. Using berberine chloride as an inhibitor, the activity of sortase A was investigated with the substrates of LPETG-EGFP protein, and compared to Dabcyl-QALPETGEE-Edans. A high yield of sortase A was achieved by inducing 1.0 mmol/L IPTG at 28 °C for 6 h. The intensity of green fluorescence of substrates displayed on the yeast surface was increased over time, while the stability was decreased slightly. Both fluorescence spectrophotometery and flow cytometry were fit for detection because of their high sensitivity. We utilized two different substrates of sortase A to investigate sortase A activity, which resulted in the increase of fluorescence intensity with respect to the increased time of growth. However, the method with Dabcyl-QALPETGEE-Edans as its substrate was more robust. Thus, the method described in this paper is a simple and cheap method which is very suitable for high-throughput analysis, but the conventional method is much more sensitive. The method described in this paper is expected to lead to large-scale screening of sortase A inhibitors which can be used to decrease the risk of drug resistance development.

## 1. Introduction

The development in the last 75 years of antibiotics that specifically target bacteria cells has been associated with cell death and the development of drug resistance is always advantageous to the microorganism [1]. The emergence of multidrug-resistant bacterial strains has limited and continues to limit the clinical efficacy of most of the currently marketed antibiotics, and threatens human life. Antibiotics have disarmed pathogenic bacteria rather than kill them, and, therefore, they have exerted low selective pressure to promote the development of antibiotic resistance [2]. Interfering with adhesion might be an efficient strategy for the prevention or treatment of bacterial infections, because the adhesion to the host tissue happens in the first step [3,4]. Exploring alternative targets not associated with bacterial growth and cell death, but rather related to virulence factors, could lead to the development of antivirulence drugs. Moreover, as opposed to conventional antibiotics, in response to which the evolution of resistance by the pathogens was advantageous and nearly unavoidable, in the case of antivirulence agents, the resulting resistance is potentially costly and therefore less probable [5].

Sortase A (SrtA) is a membrane-bound cysteine transpeptidase that is in charge of catalyzing the covalent anchoring of surface proteins to the Gram-positive bacterial cell wall. These surface proteins play critical roles in bacterial adhesion and invasion of host tissues, biofilm formation, and immune evasion by inhibition of opsonization and phagocytosis [3]. Thus, SrtA constituted an ideal target for the development of new anti-virulence agents, as SrtA is required for the Gram-positive pathogenesis of many different bacterial infections. Proteins destined for cell wall anchoring are directed for secretion by their N-terminal signal sequence and the Sec pathway [6], which consists of a pentapeptide motif (LPXTG (leucine, proline, any amino acid, threonine and glycine)) at the carboxyl terminus [7]. Secretion of the LPXTG proteins are recognized by the membrane-bound SrtA and cleaved between the Thr and Gly residues, and then anchored to the cell wall [7].

Research efforts are being made into their development as anti-virulence drugs for the alternative or complementary treatment of infectious diseases in the near future, thus offering a solution to the antibiotic resistance problem. Thousands of compounds have been developed into lead candidates for antivirulence drugs targeting SrtA. However, most of these compounds have only been used to model the action of the enzyme, and they either contain low activity, lack specificity, or display undesirable structural features, while others have provided promising hit compounds for the development of novel anti-virulence agents. These compounds could be divided roughly into three classes: (1) synthetic small molecules, such as methyl(2E)-2,3-bis(4-methoxyphenyl)prop-2-enoate [8], diaryacrylonitriles [8] and (Z)-3-(2,5-di methoxyphenyl)-2-(4-methoxyphenyl) acrylonitrile (DMMA) [9]; (2) natural products, such as maltol-3-*O*-(4′-*O*-*cis*-*p*-cumaroyl-6′-*O*-(3-hydroxy-3-methylglu-taroyl)-β-glucopyranoside [10], bis(indole) alkaloids [11], and flavonoids [12]; and (3) the substrate-derived peptides, such as vinyl sulfone [13], peptidomimetics [14], and threonine analogues [15]. The efficacy of the most promising inhibitors needs to be further evaluated for in vivo models of infection, in order to select compounds eligible for the treatment of bacterial infections in humans [16,17].

Research efforts are being made into their development as anti-virulence drugs for the alternative or complementary treatment of infectious diseases in the near future, thus offering a solution to the antibiotic resistance problem. Thousands of compounds have been developed into lead candidates for antivirulence drugs targeting SrtA. However, most of these compounds have only been used to model the action of the enzyme, and they either contain low activity, lack specificity, or display undesirable structural features, while others have provided promising hit compounds for the development of novel anti-virulence agents. These compounds could be divided roughly into three classes: (1) synthetic small molecules, such as methyl(2E)-2,3-bis(4-methoxyphenyl)prop-2-enoate [8], diaryacrylonitriles [8] and (Z)-3-(2,5-di methoxyphenyl)-2-(4-methoxyphenyl) acrylonitrile (DMMA) [9]; (2) natural products, such as maltol-3-*O*-(4′-*O*-*cis*-*p*-cumaroyl-6′-*O*-(3-hydroxy-3-methylglu-taroyl)-β-glucopyranoside [10], bis(indole) alkaloids [11], and flavonoids [12]; and (3) the substrate-derived peptides, such as vinyl sulfone [13], peptidomimetics [14], and threonine analogues [15]. The efficacy of the most promising inhibitors needs to be further evaluated for in vivo models of infection, in order to select compounds eligible for the treatment of bacterial infections in humans [16,17].

Several methods had been developed for the identification and characterization of new SrtA inhibitors. These include the screening of natural products or small compound libraries [18,19], as well as virtual screening [20,21]. Both approaches have been successfully used and have identified several SrtA inhibitors to date. High Throughput Screening (HTS) has been widely used in the search of new and potent SrtA inhibitors, as it allows the identification of active molecules among thousands of screened compounds. The activity of SrtA is measured by monitoring the cleavage of a fluorescence resonance energy transfer substrate, such as peptide Abz-LPETG-Dnp. However, the use of substrates of fluorescent peptides was frustrated largely by the high price of substrates.

Microbial cell-surface engineering has a wide range of biotechnological and industrial applications which include: live vaccine development, screening-displayed peptide libraries, bio-absorbents for the removing of harmful chemicals and heavy metals, and for biosensor developments [22,23]. It has been reported that certain natural SrtA inhibitors (berberine chloride, psammaplin A1, etc.) can inhibit *Staphylococcus aureus* cell adhesion to fibronectin (LPXTG protein) via fibronection-binding protein, resulting in success in reducing the infection rate [3,24]. In contrast to prokaryotic display technique, the yeast display technique has stark advantages, including (1) producing a soluble and functional protein; and (2) obtaining results at high densities without obtaining unwanted specific proteins via centrifugation [25]. These advantages are very helpful upon harvesting fusion proteins which are extremely difficult to purify and so are expensive to purchase [26]. Thus, the yeast display system is expected to be of great interest for further biotechnological applications, and it is suitable to display SrtA substrates to reduce costs.

To overcome these obstacles, we have established a new high throughput technique of high efficiency and low cost for the screening of inhibitors of SrtA. Firstly, we optimize SrtA expression conditions including induction time, induction temperature and induction concentration of IPTG. Then, the enhanced green fluorescence (EGFP) change of its substrates on the surface of *P. pastoris* over time was detected by flow cytometry and fluorescence spectrophotometry. Finally, using berberine chloride for positive control, the yeast strain displaying the LPXEG motif was mixed and interacted with SrtA or/and inhibitors to investigate the method of screening inhibitors of SrtA.

## 2. Materials and Methods

### 2.1. Strains and Media

The *S. aureus* strain (ATCC6538) was cultivated in LB medium. The *E. coli* Top10 (Invitrogen, Carlsbad, CA, USA) was used for vector manipulation and propagation. The *E. coli* DE3(BL21)/pTRX-srtA [27], was constructed in our laboratory and cultivated in LB medium, which was added ampicillin at 100 μg/mL as a marker. The *P. pastoris* GS115 strain (Invitrogen, Carlsbad, CA, USA) and the yeast-displayed vector pKFS [28] were used to display the LPETG-EGFPs. *P. pastoris* GS115 was cultivated in MD (1.34% (w/v) yeast nitrogen base, 6 × 10^−5^% biotin, 1% (w/v) dextrose), YPD (1% yeast extract, 2% peptone, 2% dextrose), BMGY (1% yeast extract, 2% peptone, 100 mM potassium phosphate, pH 6.0, 1.34% YNB, 4 × 10^−5^% Biotin, 1% glycerol) and BMMY (1% yeast extract, 2% peptone, 100 mM potassium phosphate, pH 6.0, 1.34% YNB, 4 × 10^−5^% Biotin, 1% methanol) medium.

### 2.2. Optimization of Expression Condition of SrtA

SrtA was induced according to a previously documented procedure [27] from the overexpression *E. coli* DE3(BL21) harboring plasmid pTRX-srtA. *E.coli* DE3/ pTRX-srtA were induced expressed in the gradual induction temperature (22 °C, 28 °C, 37 °C and 40 °C), induction time (4 h, 5 h, 6 h and 7 h) and isopropyl β-D-thiogalactoside (IPTG) concentration (0.25 mmol/L, 0.5 mmol/L, 1 mmol/L, and 2 mmol/L). Other conditions were the same as in the previously documented procedure. The induction cells were detected by SDS-polyacrylamide gels (SDS-PAGE). SrtA was gained after purification by affinity chromatography, preserved by vacuum freeze drying at −20 °C.

### 2.3. Construction of pKFS/LPETG Vector and Transformation

**Figure 1:**
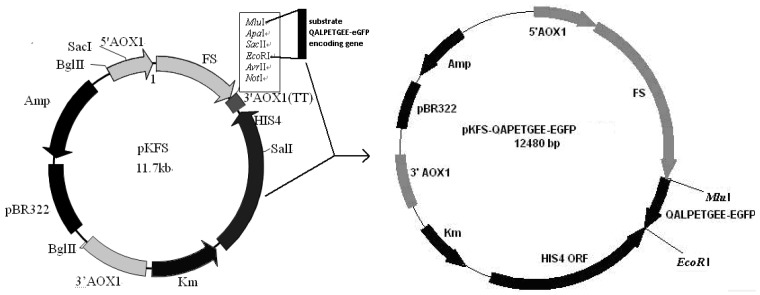
Construction of plasmid pKFS-LPETG.

To allow expression of LPETG-EGFP, its coding sequence was amplified using oligonucleotides P1: 5’-CCCACGCGTATGCAAGCTTTGCCTGAAACTGGTGAAGAAGGAGGAATTGGAATTGCTC-3’ and P2: 5’-CGCGAATTCTTACTTGTACAGCTCGTCCATG-3’, resulting plasmid vector was named pKFS-LPETG. MluI and EcoRI were used to linearize *P.pastoris* vector (Figure 1). The constructed pKFS-LPETG and pKFS were linearized and transformed into *P.pastoris* GS115 using the lithium acetate method [29]. The transformants GS115/ pKFS-LPETG and GS115/ pKFS were obtained by MD selective medium.

### 2.4. Yeast Culture

A single transformant was inoculated and kept according to the previously procedure [28]. The products were assayed by flow cytometry (Beckman-Coulter, Fullerton, CA, USA) with a total number of events of 5000 cells for each sample, and this data was analyzed using EXP032 software. The same products were determined by fluorescence spectrophotometry at excitation and emission wavelengths of 488 nm and 513 nm. Flow cytometry results were compared to the fluorescence spectrophotometry data in order to investigate the sensitivity and efficiency of the method.

### 2.5. Fluorescence Assay

SrtA activity was detected by quantifying the fluorescence intensity base on the cleavage of the yeast-surface-displayed LPETG protein. Reactions were performed in a total volume of 5 mL and the culture and berberine chloride or control treatment was added. A 150 μL volume of GS115/pKFS-LPETG-EGFP (OD600 = 1.0) or the same amount of GS115/pKFS (as a negative control) was added to each aliquot, 5 μM SrtA, and this was transferred to an Eppendorf tube. The mixture was tested every 30 min for 2 h by fluorescence spectrophotometry. Then, decreasing concentrations starting from 1 μg/mL to 30 μg/mL of berberine chloride (a well-known sortase inhibitor was purchased from Kaitong chemical Co., Ltd., Tianjin, China) was added and the effect was investigated according to the activity of SrtA, and the results were compared to the substrates of Dabcyl-QALPETGEE-Edans [30]. In this way it was possible to compare any differences in binding of the cells to SrtA due to differences in inhibitor concentrations treatment and cell growth.

## 3. Results

### 3.1. Optimization of pTRX-srtA Vector’s Expression

**Figure 2:**
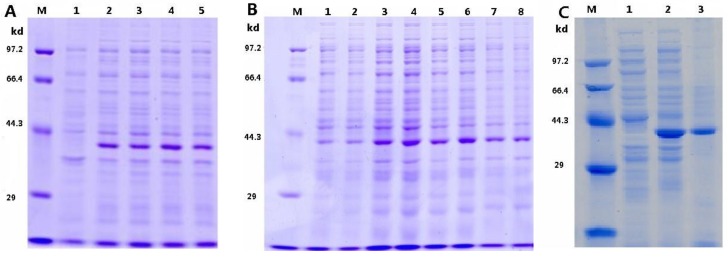
Expression of pTRX-srtA. (**A**) Effects of induce time on pTRX-srtA expression. M: Protein molecular mass standards; 1: Lysate of pTRX-srtA transfectant without induction; 2–5: Lysate of pTRX-srtA transfectant induced with 1.0 mmol/L IPTG at 37 °C for 4 h, 5 h, 6 h and 7 h, respectively; (**B**) Effects of IPTG concentration and induction temperature on pTRX-srtA expression. M: Protein molecular mass standards; 1–4: Lysate of pTRX-srtA transfectant induced at 37 °C for 6 h with different IPTG concentration of 0.25 mmol/L, 0.5 mmol/L, 1.0 mmol/L, and 2.0 mmol/L, respectively; 5–8: Lysate of pTRX-srtA transfectant induced with 1 mmol/L IPTG for 6 h, at the temperature of 22 °C, 28 °C, 37 °C and 40 °C; (**C**) Purification of pTRX-srtA proteins. M: Protein molecular mass standards; 1: Lysate of pTRX-srtA transfectant without induction; 2: unpurification of pTRX-srtA transfectant with 1.0 mmol/L IPTG at 28 °C for 6 h; 3: purification of pTRX-srtA transfectant with 1.0 mmol/L IPTG at 28 °C for 6 h.

The expression levels of pTRX-srtA vector under different induction times (in Figure 2A), IPTG concentrations and induction temperatures was examined by SDS-PAGE (Figure 2B). The results indicated that the expression of pTRX-srtA vector was significantly influenced by the induction temperature, time, and IPTG concentration. The optimal induction time was determined to be 6 h, induction temperature was 28 °C and IPTG concentration was 1.0 mmol/L. Prolonged induction times (from 4 h to 6 h) resulted in an increase in the yield of SrtA, while shorter induction times (from 6 h to 7 h) resulted in a reduction. As IPTG-induction concentration was increased, the yield of SrtA was increased. It increased slowly until IPTG-induction concentration exceeded 1.0 mmol/L. When the SrtA was induced at the temperature of 22 °C, 28 °C, 37 °C and 40 °C, the expression gave a sharp band under the condition of 28 °C. Under the optimized expression conditions, the expressed protein accumulated up to about 90% of total bacterial protein after purification using affinity chromatography (in Figure 2C). These results demonstrated that the pTRX-srtA vector was efficiently expressed in *E. coli* BL21 (DE3) under the optimized expression conditions. The purity product was collected by centrifugation and then dried by vacuum freezing in a dryer to obtain the solid powder of the neutral SrtA.

### 3.2. Analysis of the EGFP Protein Expressed on Cell Surface of P. pastoris

**Figure 3:**
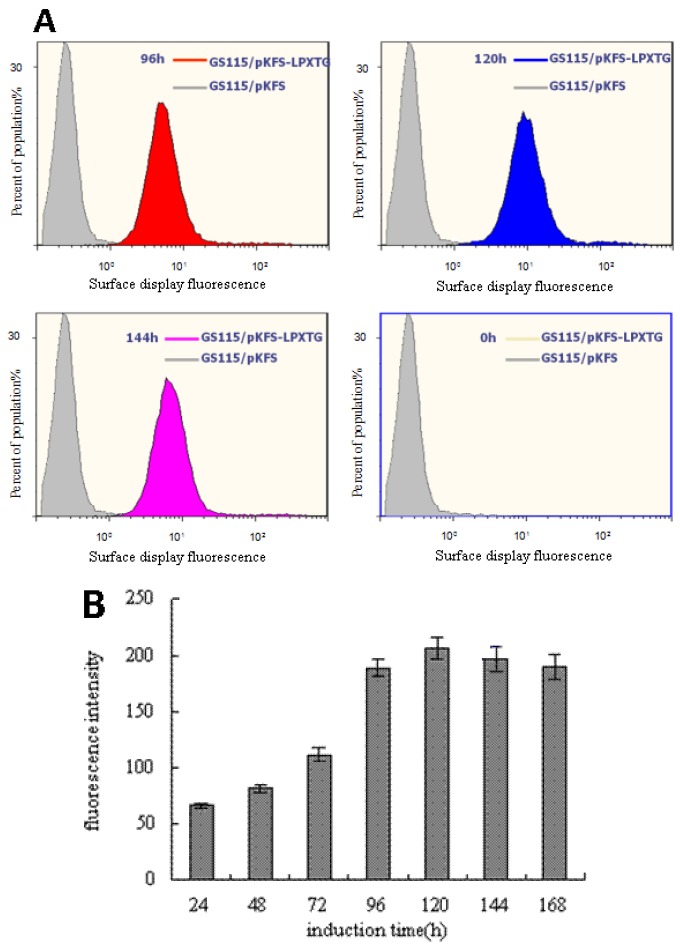
Detection of LPXTG-EGFP displayed on yeast surface. (**A**) Flow cytometry detection of LPXTG-EGFP displayed on yeast surface. (**B**) Fluorescence spectrophotometry detection of LPXTG-EGFP displayed on yeast surface.

Flow cytometry histograms were depicted (in Figure 3A). The mean fluorescence signal of LPXTG-EGFP proteins was at several different times, 0 h (yellow), 96 h (red), 120 h (blue) and 144 h (pink), as the control of GS115/pKFS cells (gray) had poor fluorescence signal at the same time. The time courses of LPXTG-EGFP proteins were detected by fluorescence spectrophotometry (in Figure 3B), and depicted the mean fluorescence signal of the yeast-displayed LPXTG-EGFP proteins. The green fluorescence signal that emitted from GS115/pKFS-LPETG was clearly detected by flow cytometry (in Figure 3A) and fluorescence spectrophotometry (in Figure 3B), while hardly any green fluorescence signal emitted from GS115/pKFS. Therefore, the results showed that the fusion proteins were expressed at a high level, and the total number of EGFP proteins increased up to 120 h and then slightly decreased up to 168 h. Furthermore, it is shown that both methods could be used to detect SrtA activity. Compared with other methods, the biggest advantage of spectrophotometry is its affordability.

### 3.3. Detection of SrtA Activity

The SrtA activity assay is based on the fact that the fluorescence intensity will change as the LPETG-EGFP on the yeast surface are cleaved by SrtA. An inhibitor would affect the SrtA in such a way that they will keep the -EGFP on the yeast surface and will not be cleaved by SrtA. By investigating treated and untreated samples over time and by investigating the differences in cleaving, the idea is that it would be possible to study an inhibition.

**Figure 4:**
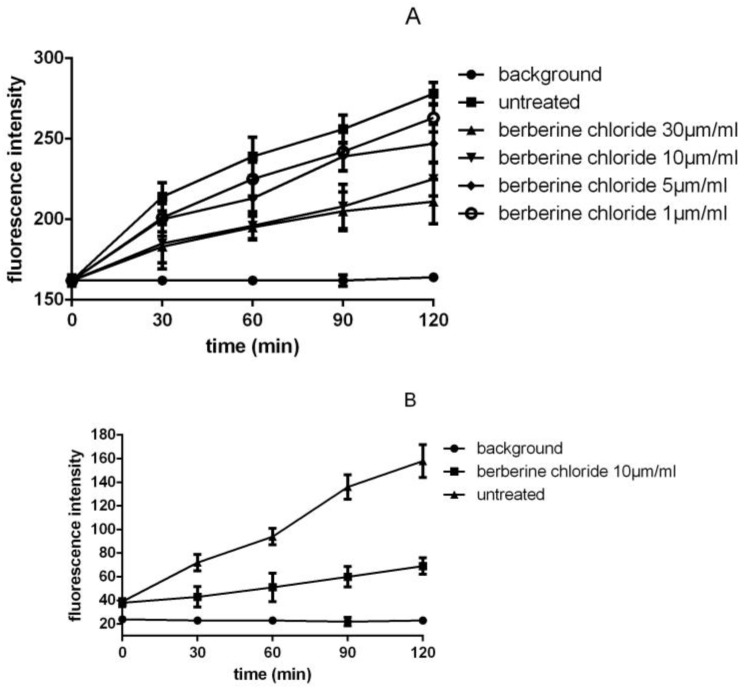
Fluorescence spectrophotometry detection of SrtA activity. (**A**) Fluorescence spectrophotometery detection of SrtA interaction with LPXTG-EGFP in time. (**B**) Fluorescence spectrophotometry detection of SrtA interaction with Dabcyl-QALPXTGEE-Edans in time.

The results of the fluorescence spectrophotometry assay in Figure 4A show that the fluorescence changes are linear by the time that the yeast surface display of substrate LPETG-EGFP interacted with SrtA. Berberine chloride was added in the reactions using different concentrations to investigate the potential role of the inhibitor. Also, a high linearity is seen in the results and a general trend in the difference between the control and the sample with inhibitor was recorded. Before investigating the inhibition of SrtA, it is crucial that a successful assay for the untreated sample is developed. The fluorescence intensity of Dabcyl-QALPXTGEE-Edans of the positive control group was diminished by fluorescence spectrophotometry investigation with 350 nm for excitation and 495 nm for recordings shown in Figure 4B. All the results indicated that the control and the sample with inhibitor increased with respect to increased time of growth which was fitted for the screening of inhibitors. However, the positive control was more robust.

## 4. Discussion

The emergence of Gram-positive bacteria with intermediate or full resistance to antibiotics has become a major public health threat [31]. The bacteria need to be pathogenic to develop an infection, and they need to interact with the host. Gram-positive bacteria mediate this interaction via a special mechanism involving anchoring of proteins present in the cell membrane to the peptidoglycan [32]. These proteins are believed to be essential for the survival of the bacteria during the infection [33]. A housekeeping enzyme called SrtA is responsible for this mechanism and might be a suitable target for inhibition to prevent the bacterial infection developing [34].

To be able to investigate the activity of SrtA and its potential inhibitors, different approaches were investigated, including FRET assay, labeling of bacterial cells, a fibronectin-binding protein assay, and so on. FRET assays [18,19] have been created and these assays turned out to be a good starting point to study inhibition, but due to the complex manipulation and the high costs of synthesized substrates the assay does not seem to be convenient or economical enough for the investigation of inhibitors. Both the labeling of bacterial cells [24] and the fibronectin-binding protein assay provided an advantage for investigating the SrtA in its natural environment. Unfortunately, although the protocol was adjusted in a number of different ways, the results of the assays indicated that the assay does not seem to be robust and sensitive enough for the investigation of inhibitors.

In this study, to optimize the induction condition of SrtA expression in *E. coli* DE3(BL21) and catalyze the LPXTG motif of the substrate which displays on the surface of *P. pastoris* for detecting the activity of SrtA and screening its inhibitors, the green fluorescence intensity of LPETG-motif displaying on the surface of *P. pastoris* was increased, and it was detected by flow cytometry (in Figure 3A) and fluorescence spectrophotometry (in Figure 3B). The results are a clear indication that fluorescence intensity change of the LPETG-motif becomes clearer with time. Moreover, these LPETG-motifs were correctly folded under the conditions studied and rarely disturbed by other cell-surface proteins. Both methods could reflect the relationship between the change of fluorescence and the catalysis of SrtA well. These results also indicate that *P. pastoris* displaying the LPETG-motif may constitute immobilized and purified substrates which are widely applicable to detecting SrtA activity. In earlier studies, when SrtA or the substrates were expressed on the cell-surface, this would lead to a barely efficient interaction with each other as the interaction might be broken by the interference of the other cell-surface proteins, etc. The yeast-display system method has the following advantages: easy manipulation of gene clone, protein expression, and cell concentration, while the maximal difficulty was finding a different protocol to achieve a robust assay that is sensitive enough for investigation of SrtA.

With the aim of reaching a more robust assay, the protocol was adjusted in various ways. The routine SrtA assay is based on the production of enough native or synthesized substrates, but the complicated purification process and the high expenses greatly limit the screening for inhibitors. In the present study, a novel SrtA method was developed by applying the yeast-displaying substrates directly into the SrtA detection system instead of soluble protein substrates. The displayed- substrates were successful in the detection of SrtA during the time of the project. The results were confirmed by fluorescence spectrophotometry. All the results indicated that the control and the sample with the inhibitors (different concentration of berberine chloride) were increased with respect to growth, which was fitted for screening inhibitors, but the positive control was less variant. Even though the routine method was much more sensitive, the yeast-displaying substrates method was easier and cheaper, making it well suited to large-screening inhibitors.

Recently, cell surface engineering has made significant progress and has been exploited to represent many new functions. After improving the operation process and increasing the sensitivity, SrtA activity could be checked with the yeast-displaying substrates by flow cytometry and fluorescence spectrophotometry. The yeast-displaying substrates could totally replace recombinant proteins or synthesized substrates. The novel SrtA detection could be applied further to form a new protein-free LPXTG-motif production method which would avoid the purification of recombinant proteins and refrain from relying on there being enough catalytically recombinant protein in the traditional LPXTG-motif production. The course of LPXTG-motif production would take much less time from gene cloning to SrtA assessment than the traditional way. Presumably, the yeast-displaying substrates could interact with SrtA directly, thereby enabling them to behave more efficiently. It would accelerate the new, high-throughput, protein-free SrtA assay, which is promising and means that it could even replace the conventional method.

## 5. Conclusions

The method that was described in this paper is a simple and cheap method, which is very suitable for high throughput analysis. However, the traditional method is much more sensitive. This approach is expected to lead to large-scale screening of SrtA inhibitors. It can be used to decrease the risk of drug resistance development.
